# Evaluation of urinary kidney injury molecule-1 in cats with chronic kidney disease

**DOI:** 10.1177/1098612X251314778

**Published:** 2025-04-15

**Authors:** Matthew Kornya, Alice Defarges, Dorothee Bienzle

**Affiliations:** 1 Department of Clinical Studies, Ontario Veterinary College, University of Guelph, Guelph, ON, Canada; 2 Department of Pathobiology, Ontario Veterinary College, University of Guelph, Guelph, ON, Canada

**Keywords:** Biomarker, nephrology, renal, laboratory medicine

## Abstract

**Objectives:**

Kidney injury molecule 1 (KIM-1) is a transmembrane glycoprotein on proximal renal tubular epithelial cells that is increased in the urine of cats with acute kidney injury. The utility of measuring urine KIM-1 in cats with chronic kidney disease (CKD) and the relationship with International Renal Interest Society (IRIS) stage are unknown. The objectives of this study were to determine the distribution of KIM-1 concentrations in cats with different stages of CKD and investigate the relationship between urine KIM-1 and urine specific gravity (USG), urine protein and serum urea, creatinine, phosphorus, potassium and symmetric dimethylarginine concentrations.

**Methods:**

A total of 74 cats with CKD were recruited prospectively from a first-opinion feline-only practice. Blood and urine samples were collected from all cats. The stage of CKD was determined as per IRIS guidelines. Urine KIM-1 concentration was determined with a previously validated lateral flow assay. KIM-1 was reported as a test:control ratio. The distribution of KIM-1 values in cats with CKD was determined, and the correlation between KIM-1 and other determinants of renal function was calculated. Urine KIM-1 was normalized to a USG of 1.035 and the analysis was repeated.

**Results:**

Cats with CKD had a median urine KIM-1 value of 0.1544 (range 0.038–0.540). The median KIM-1 values in cats with IRIS stage 1, 2, 3 and 4 CKD were 0.152 (range 0.113–0.512), 0.165 (range 0.038–0.540), 0.150 (range 0.037–0.448) and 0.140 (range 0.067–0.448), respectively. There were no differences in urine KIM-1 values relative to IRIS stage. Urine KIM-1 values were correlated with USG (*r*^ 2^ = 0.482; *P* = 0.005). An analysis of KIM-1 values normalized to USG resulted in similar findings.

**Conclusions and relevance:**

Urine KIM-1 values in cats with CKD were similar to those previously described in healthy cats. There was a moderately strong correlation between urine KIM-1 concentration and USG. Sequential measurement of KIM-1 in cats with progressive CKD may be informative.

## Introduction

Chronic kidney disease (CKD) is among the most common diseases of cats, affecting approximately 30–50% of cats aged over 10 years.^1^ Acute kidney injury (AKI) typically occurs independently from CKD and is due to exposure to toxins, infections, upper or lower urinary tract obstruction, or idiopathic causes.^2,3^ AKI may also occur in animals with CKD, in which case it is often termed ‘acute-on-chronic kidney disease’ (ACKD).^2,3^ Despite advances in the diagnosis of AKI and ACKD, the early detection of AKI and differentiation of AKI from ACKD remain challenging.^4,5^

The diagnosis of kidney disease may be accomplished either through measuring markers of renal function (ie, surrogates of glomerular filtration rate such as serum creatinine [sCr], urea or symmetric dimethylarginine [SDMA] concentration) or kidney injury (ie, urine kidney injury molecule-1 [KIM-1], cystatin, neutrophil gelatinase-associated lipocalin [NGAL], etc).^6^ Markers of renal function are, in general, less sensitive to AKI than markers of injury since renal function must be substantially impaired to see increases in serum markers. Aside from AKI, non-renal chronic diseases may also affect the serum and urine concentration of markers of renal injury (eg, cystatin C or fibroblast growth factor-23 in hyperthyroidism, NGAL with systemic inflammation).^4,5^

KIM-1 is a type I transmembrane glycoprotein that facilitates the removal of apoptotic debris from the tubular lumen and promotes cell-to-cell and cell-to-matrix adhesion.^7,8^ It is rapidly and markedly upregulated within regenerating epithelial cells in the S3 segment of the proximal tubules after ischemic, toxic, septic, hypovolemic, hypotensive or transplant-related renal injury.^9^ KIM-1 is expressed at low levels in healthy feline and human kidney tissue, likely associated with normal turnover of proximal tubular cells.^5,10^

During renal tubular injury, an extracellular portion of KIM-1 is released into the urine, allowing for non-invasive detection and monitoring. Studies in multiple species, including cats, have shown that injured renal tubules upregulate and shed KIM-1 into the urine before clinically relevant increases in sCr.^10–13^

A lateral flow assay (LFA) has been validated for the determination of urine KIM-1 concentration in healthy cats.^12^ The stability of KIM-1 over time and at different temperatures is high, and a reference interval (RI) for KIM-1 in healthy cats has been described.^10–12,14,15^ The assay is able to detect KIM-1 at concentrations as low as 3 ng/ml. However, there is limited knowledge about KIM-1 concentration in cats with CKD.

The purpose of this study was to evaluate the concentration of KIM-1 in the urine of cats with CKD and its association with other markers of renal disease.

## Materials and methods

Samples were recruited prospectively from cats diagnosed with CKD in a primary care feline-only practice. Testing was performed on residual urine samples left over after routine diagnostic testing. Cats were included if their sCr or SDMA concentration was above the RI while adequately hydrated and the urine specific gravity (USG) was decreased (<1.035) without an identifiable cause other than CKD; for International Renal Interest Society [IRIS] stage 1 cats, there were ultrasonographic changes consistent with CKD (renal asymmetry, loss of corticomedullary definition, hyperechoic cortices, mineralization) as well as a USG <1.035. Cats included were required to have persistent changes over at least two profiles in a 6-month period. Cats were excluded if there had been evidence of AKI in the past 2 months, such as an increase in sCr of >27 µmol/l, a diagnosis of upper or lower urinary obstruction, exposure to known nephrotoxins (including non-steroidal anti-inflammatory drugs or diuretics), general anesthesia or another procedure potentially associated with hypotension, or weight loss of >10% body weight. The stage of CKD was determined based on IRIS guidelines using sCr and SDMA.^16^

All blood samples were collected by either jugular or medial saphenous venipuncture. All urine samples were collected by cystocentesis. Age, breed, sex, sCr, urea, SDMA, phosphorus, potassium, USG, urine dipstick protein, urine pH and IRIS stage were recorded for each patient. All biochemical testing and urinalyses were performed at a commercial reference laboratory (IDEXX Labs, Markham, Ontario, Canada). Systemic blood pressure and urine protein:creatinine ratio (UPC) were not consistently available in all cats and as such were not included in the analysis. All cats had a full urinalysis with sediment evaluation. Cats were excluded if there was gross hematuria, bacteriuria, crystalluria, clinical signs of a urinary tract infection or growth of any bacteria on culture other than *Enterococcus*.

In all cats, urine KIM-1 concentrations were determined using the feline KIM-1 LFA (Bioassay Works, Ijamsville, MD, USA) using 20 µl of urine diluted in a sample buffer, with 100 μl of this sample being used in the assay. This protocol is similar to one previously described, but with optimization performed during development involving a larger volume of urine.^12^ In this assay, the binding of gold nanoparticle-linked antibodies to feline KIM-1 is measured with an optical reader with a radiofrequency identification (RFID) card and reported as an optical density. The value of the test result relative to the control result is generated in each assay. The control strip is a proprietary solution containing a molecule known to bind IgG. The use of this ratio to a standardized positive control accounts for variations in environmental factors that affect immunoassays. All KIM-1 assays were run in house within 1 h of sample collection. Samples were held in the refrigerator before analysis of uncentrifuged samples.

Datatab (DATAtab e.U. Graz, Austria; https://datatab.net) was used for all statistical analyses. Multiple linear regression analyses were performed to investigate potential correlations between KIM-1 and the other variables. KIM-1 results from cats in each group of IRIS stage CKD were tested for normality using the Shapiro–Wilk test and Q–Q plot evaluation. The Kruskal–Wallis test with the post-hoc Dunn’s test was used to determine if there was a significant difference in KIM-1 between groups.

The Kruskal–Wallis test with post-hoc Dunn’s test was also used to determine if there was a significant difference in age, sex or breed between IRIS stages.

KIM-1 was normalized to a USG of 1.035 for all patients to adjust for differences related to concentrating ability (nKIM-1 = KIM-1/USG × 1.035). The analysis was repeated as above using the nKIM-1 value, with the exclusion of USG from this analysis.

## Results

Samples from 74 cats with CKD and a median age of 15 years (range 3–21) were obtained. In total, 50 cats were domestic shorthair cats, with other breeds including domestic mediumhair (n = 5), domestic longhair (n = 5), Persian (n = 2), Devon Rex (n = 2), Russian Blue (n = 1), Siamese (n = 3), Ragdoll (n = 2), Snowshoe (n = 1), Manx (n = 1), Birman (n = 1) and Scottish Fold (1). Detailed information is available in Table 1. Because of the small number of non-domestic breeds, cats were classified as domestic (n = 60) or purebred (n = 14) for analysis.

**Table 1:** Signalment of cats with chronic kidney disease

IRIS stage	Number of cats	Median (range) age (years)	Sex
1	13	15 (9–18)	MN 4; FS 9
2	31	15 (4–21)	MN 13; FS 18
3	21	15 (3–19)	MN 10; FS 11
4	9	12 (3–17)	MN 9; FS 0

Data are n or median (range)

FS = female spayed; IRIS = International Renal Interest Society; MN = male neutered

Age was not associated with IRIS stage (*P* = 0.123); but older age was associated with lower sCr (*P* = 0.005). Male cats had a higher sCr than females (mean 357 µmol/l [range 125–>1200] vs 210 µmol/l (range 99–409); *P* = 0.004) and higher median IRIS stage (3 vs 2; *P* = 0.011).

KIM-1 values were normally distributed for cats with IRIS stage 1 CKD, right skewed for IRIS stages 2 and 3, and borderline normal for IRIS stage 4 (see Table 1 for the number of cats in each group).

**Figure 1:**
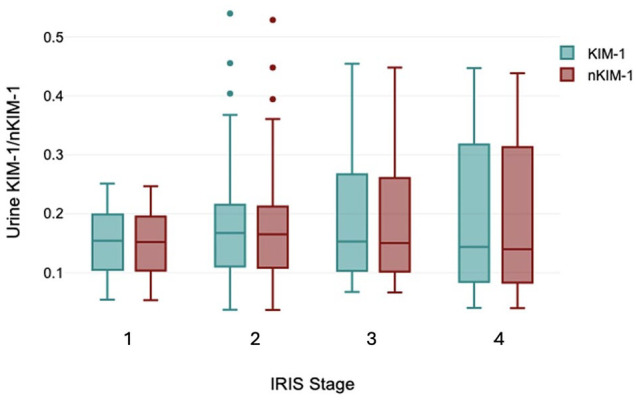
Box and whisker plots of urinary kidney injury molecule-1 (KIM-1) and urine specific gravity-normalized KIM-1 values (optical density of lateral flow assay relative to a control result) in cats with chronic kidney disease stratified by International Renal Interest Society (IRIS) stage. Solid line = median; box = quartiles; whiskers = maximum and minimum values; dots = outliers. There were no statistically significant differences between groups. All values are within the previously defined reference interval for healthy cats. CKD = chronic kidney disease; IRIS = International Renal Interest Society; LFA = lateral flow assay; USG = urine specific gravity

Urine KIM-1 for all cats with CKD combined had a median value of 0.155 (range 0.038–0.540). Median KIM-1 values were 0.152 (range 0.113–0.512), 0.165 (range 0.038–0.540), 0.150 (range 0.037–0.448) and 0.140 (range 0.067–0.448) in cats with IRIS stage 1, 2, 3 and 4 CKD, respectively (Figure 1). There were no significant differences between the IRIS stage groups (*P* = 0.424).

nKIM-1 for all cats with CKD combined had a median value of 0.155 (range 0.041–0.549). Median nKIM-1 values were 0.155 (range 0.052–0.259), 0.197 (range 0.041–0.549), 0.106 (range 0.072–0.45) and 0.155 (range 0.041–0.466) in cats with IRIS stage 1, 2, 3 and 4 CKD, respectively. There were no significant differences in nKIM-1 between the IRIS stage groups (*P* = 0.421).

There were no significant associations between breed (*P* = 0.209), age (*P* = 0.204) or sex (*P* = 0.386) and urine KIM-1 concentration. There were likewise no significant associations between breed (*P* = 0.482), age (*P* = 0.215) or sex (*P* = 0.393) and nKIM-1.

Multiple regression analysis showed that urine KIM-1 was moderately correlated with USG (Table 2) but not with sCr, urea, SDMA, potassium, phosphorus, urine dipstick protein or urine pH. The *r*^2^ value for the correlation of KIM-1 and USG was 0.482.

**Table 2:** Multiple regression coefficients and *P* values for the effect of chronic kidney disease-associated parameters on urinary kidney injury molecule-1 (KIM-1), and on KIM-1 normalized to USG (nKIM-1)

	KIM-1		nKIM-1	
	Standardized coefficient	*P* value	Standardized coefficient	*P* value
Creatinine	0.38	0.269	0.18	0.701
Urea	−0.37	0.238	−0.52	0.151
SDMA	0.23	0.327	0.31	0.221
Phosphorus	−0.37	0.075	0.20	0.360
Potassium	0.03	0.795	−0.06	0.640
USG	0.35	0.011*	n/a	n/a
pH	−0.18	0.135	−0.1	0.480
Protein trace	0.03	0.822	0.07	0.647
Protein 1+	0.14	0.247	0.28	0.048*
Protein 2+	0.2	0.132	0.23	0.968
Protein 3+	−0.03	0.835	0.23	0.107

Data are n or median (range)

* Statistically significant values

K = potassium; n/a = not applicable; Phos = phosphorus; SDMA = symmetric dimethylarginine; USG = urine specific gravity

Multiple regression analysis showed that urine nKIM-1 was associated with the presence of 1+ proteinuria on dipstick (*P* = 0.048) but not with other values (Table 2).

## Discussion

In this study, urine KIM-1 concentrations were not significantly different in cats with different stages of CKD. In the light of current understanding of the pathophysiology of CKD, ongoing acute injury of proximal tubules is either not a feature or only a minor feature of stable CKD, although this has been challenged in some recent publications.^17^ Rather, CKD in most instances is thought to reflect reduced nephron number and therefore limited filtration and reabsorption capacity, which may remain relatively static over months.^3^ In a recent study of healthy cats, the RI for urine KIM-1 was 0.02–0.68, a range that would encompass the values found in cats with CKD in this study.^15^ The mean urine KIM-1 value in that study was 0.354, compared with 0.183 in the current study. Although this may suggest that urine KIM-1 is lower in cats with CKD than in healthy cats, it may also reflect the inability of cats with CKD to fully concentrate urine or a reduced ability to regenerate damaged epithelial cells. Nonetheless, the overlap in raw values implies that KIM-1 may be similar in healthy cats and in those with CKD.

KIM-1 has been investigated in cats with AKI in several scenarios, with data suggesting increased urinary KIM-1 in both spontaneous and experimental disease.^10,12^ In the initial description of the KIM-1 LFA, cats with AKI had urinary KIM-1 that in some cases overlapped with that of healthy cats; however, the mean KIM-1 was higher in the AKI group and the maximum KIM-1 in cats with AKI was more than double the maximum value detected in the healthy group.^12^ Because of the specific proximal tubular production of KIM-1 and the finite duration of excretion in the course of AKI, it is likely that KIM-1 will not be increased in all cats with AKI; however, marked increases are likely consistent with injury. Although ideally the current study would have included a group of healthy cats, cats with CKD and cats with AKI, this was not feasible with reasonable sample sizes from a single primary care center.

In a recent study, a marked increase in markers of renal injury was reported in cats with CKD, a finding not supported by this study.^17^ In that study, urine cystatin B was used as a marker of renal injury. In dogs, urine cystatin B is predictive of progressive vs stable kidney disease, potentially since it is more likely to detect low-grade AKI or progressive nephron death.^18^ It is also possible that the discrepancy of increased urine cystatin B but not KIM-1 in urine is due to different cellular origins (pan-renal for cystatin B and specific proximal tubular segments for KIM-1), differences in stage of kidney disease or inclusion criteria, or other factors. It is also possible that the smaller sample size or a different rate of disease progression in the study concerning urine cystatin B may have contributed to the differences in the results from this study.

The results from this study suggest that urine KIM-1 is not suitable for the diagnosis of CKD, but also that increases in KIM-1 are not expected in cats with CKD. This supports the hypothesis that feline CKD does not involve significant ongoing tubular injury. As such, cats presenting to a veterinarian with increased urine KIM-1 above the RI may be more likely to have AKI than simply CKD that has progressed. As it is not currently known how much KIM-1 variation occurs in healthy cats, monitoring this value over time may have increased diagnostic yield. This may be a clinically useful feature for the differentiation of late-stage disease from an acute-on-chronic crisis; however, further research in cats with identifiable ACKD is needed to more closely define KIM-1 in this condition.

In a previous study of KIM-1 values in healthy cats, age, sex, body condition score, blood pressure, phosphorus, SDMA, creatinine, total thyroxine and mid-sagittal kidney length were not significant predictors of urine KIM-1.^15^ This agrees with the results of the current study. In both the current and the previous study of healthy cats, USG was found to be associated with urine KIM-1 concentration.

Urine KIM-1 was moderately positively correlated with USG in this and other studies.^15^ This correlation may be due to dilution of urine KIM-1 at a lower USG or may be due to decreased shedding of the protein in animals with reduced urine-concentrating ability. Normalization of KIM-1 to a standard USG had little effect on its correlation with other renal values and did not result in an association with IRIS stage. It is possible that utilization of urine creatinine rather than USG may have been a more accurate reflection of urine concentration. USG may be affected by several factors and has limited utility for the interpretation of other urine markers, such as proteinuria.^19^ However, the KIM-1 LFA is intended as a point-of-care assay, and urine creatinine is not readily available for normalization patient-side, hence USG was thought to be more practical. Urine creatinine was also not routinely measured in all patients in this study. This use of a KIM-1:creatinine ratio was slightly superior to KIM-1 concentration for the detection of AKI in humans.^13^ In humans, urine KIM-1 increases with proteinuria.^7^ However, this finding was reported in humans with diabetic nephropathy, a population that generally has more proteinuria than the relatively mild values seen in cats with CKD, and results therefore may not be comparable.^13,16^ The association of nKIM-1 with 1+ proteinuria is suspected to be spurious, as it is not physiologically plausible that nKIM-1 relates only to specific degrees of proteinuria. Further investigations, such as correlations between nKIM-1 and UPC may be warranted.

It is possible that cats with early stages of CKD that experience AKI or early nephron loss, and cats with advanced CKD that progresses rapidly, may have transiently higher urine KIM-1 than cats with stable CKD. Similar to many studies of feline CKD, most cats in this study had stable IRIS stages 2 and 3, and few had stage 1 or 4 disease. Therefore, evaluation of KIM-1 in cats with IRIS stage 1 and 4 disease may be of interest.

Since KIM-1 is released from injured renal tubular epithelium, increases in urine KIM-1 may reflect generalized injury where urine flow is maintained, or predominant contributions from a few foci of severely injured nephrons. Obstruction of urine flow by necrotic cell debris in severe injury will likely also affect the relative amount and timing of KIM-1 in urine.^10^ Neither the former nor the latter scenario was captured in this study of cats with stable CKD.^17^

## Conclusions

The results of this study indicate that neither normalized nor direct urine KIM-1 concentrations are affected by the stage of CKD and that the concentration of urine KIM-1 is similar to that in cats without CKD. While there was a moderately strong correlation of KIM-1 with USG, other markers of renal function did not correlate with KIM-1.

## Supplemental Material

Supplemental Material Biochemical, signalment and urinalysis parameters for cats with CKD.
