# Supplementary material for: Evaluation of urinary kidney injury molecule-1 in cats with chronic kidney disease
Source: J Feline Med Surg. 2025 Apr 15;27(4):1098612X251314778. doi: 10.1177/1098612X251314778 (PMC12035179; doi:10.1177/1098612X251314778)
Supplement: Supplemental Material [file sj-docx-1-jfm-10.1177_1098612X251314778.docx]

| Age (y) | Breed | Sex | KIM-1 Test | KIM-1 Cont. | KIM-1 T/C | Creat (umol/L) | Urea (mmol/L) | SDMA (ug/dL) | Phos (mmol/L) | K (mmol/L) | Urine S.G. | Urine pH | Prot. | Sediment | Urine Culture | UPCR | Blood Pressure (Doppler) |
| --- | --- | --- | --- | --- | --- | --- | --- | --- | --- | --- | --- | --- | --- | --- | --- | --- | --- |
| 18 | DSH | FS | 11.6 | 135.5 | 0.0856 | 402 | 31.9 | 30 | 2.3 | 3.5 | 1.014 | 5.5 | 2+ | Rare RBC, 0-2 WBC | n/a | 0.6 | 135 |
| 11 | DLH | MN | 15.9 | 123.6 | 0.1286 | 150 | 10.7 | 16 | 1.6 | 4.9 | 1.013 | 6.5 | 1+ | Inactive | n/a | n/a | 300 |
| 11 | DLH | MN | 51.8 | 115.8 | 0.4473 | 474 | 40.1 | 31 | 3.9 |  | 1.02 | 6 | Tr | Inactive | n/a | n/a | 110 |
| 17 | DSH | FS | 15.3 | 99.2 | 0.1542 | 99 | 12.3 | 20 | 1.2 | 3.8 | 1.015 | 6.5 | 0 | Inactive | n/a | n/a | n/a |
| 11 | DMH | FS | 44.2 | 120.2 | 0.3677 | 192 | 16.4 | 12 | 1.4 | 4.3 | 1.02 | 5 | 0 | Inactive | n/a | 0.1 | n/a |
| 17 | DSH | MN | 14.9 | 121.5 | 0.1226 | 731 | 57.9 | 43 | 3.4 | 5.1 | 1.012 | 5 | Tr | Inactive | NG | 0.3 | 140 |
| 15 | DMH | FS | 29.8 | 111.3 | 0.2677 | 266 | 12 | 21 | 1.43 | 2.5 | 1.018 | 6 | 2+ | Inactive | n/a | 1.3 | n/a |
| 13 | DSH | FS | 11 | 90.7 | 0.1213 | 163 | 12.8 | 9 | 1.3 | 3.9 | 1.029 | 6.5 | 3+ | Moderate RBCs, CaOx | n/a | n/a | n/a |
| 13 | DSH | FS | 5.6 | 123.3 | 0.0454 | 158 | 11.7 | 8 | 1.3 | 4 | 1.016 | 5.5 | 0 | Moderate RBCs | n/a | n/a | n/a |
| 10 | DSH | FS | 10.2 | 106.9 | 0.0954 | 194 | 27 | 14 | 1.5 | 4.6 | 1.012 | 6 | Tr | Inactive | NG | n/a | n/a |
| 18 | DSH | FS | 19.9 | 109.9 | 0.1811 | 163 | 19 | 17 | 2.4 |  | 1.018 | 6 | 1+ | >50 RBC | n/a | n/a | n/a |
| 16 | Devon Rex | FS | 10.2 | 116.3 | 0.0877 | 135 | 16.8 | 20 | 1.7 | 4.1 | 1.013 | 5.5 | 0 | 0-2 RBCs and WBCs | n/a | 0.3 | 130 |
| 9 | DSH | MN | 22.1 | 122.2 | 0.1809 | 125 | 8 | 20 | 1.3 | 4.6 | 1.015 | 6.5 | 0 | Inactive | n/a | n/a | n/a |
| 16 | DSH | FS | 7.2 | 117.6 | 0.0612 | 224 | 17.6 | 21 | 2.9 | 4.5 | 1.017 | 6 | Tr | Inactive | NG | n/a | 155 |
| 15 | DSH | FS | 67.5 | 125.1 | 0.5396 | 224 | 13.2 | 16 | 1.3 | 4.7 | 1.021 | 5.5 | 3+ | 0-2 RBCs, 0-2 WBCs | NG | n/a | n/a |
| 17 | DSH | MN | 9.9 | 108.3 | 0.0914 | 410 | 27.2 | 23 | 1.5 | 5.2 | 1.014 | 5.5 | Tr | Inactive | n/a | 0.3 | n/a |
| 17 | DMH | MN | 54.1 | 118.8 | 0.4554 | 143 | 13.8 | 13 | 1.6 | 4.8 | 1.017 | 5 | 1+ | Moderate RBCs, few WBCs | n/a | 0 | 120 |
| 11 | DSH | MN | 18.4 | 104.8 | 0.1756 | 126 | 13.6 | 23 | 1.7 | 5.2 | 1.018 | 6 | Tr | 2-5 WBC, 0-2 RBC | n/a | n/a | n/a |
| 3 | Scottish Fold | MN | 28.9 | 108.2 | 0.2671 | 333 | 16.7 | 36 | 2.8 | 4.7 | 1.024 | 6 | 0 | Inactive | NG | 0.1 | 120 |
| 12 | DSH | FS | 15.5 | 116.1 | 0.1335 | 142 | 16.2 | 8 | 2.2 | 5.8 | 1.018 | 6 | 3+ | Inactive | Enterococcus | n/a | n/a |
| 15 | Devon Rex | FS | 13.4 | 125.9 | 0.1064 | 130 | 16.8 | 17 | 1.6 | 3.8 | 1.018 | 5.5 | Tr | 3-5 WBCs | NG | n/a | 135 |
| 11 | DSH | MN | 12 | 116.2 | 0.1033 | 397 | 30.9 | 21 | 2.7 | 5 | 1.012 | 5.5 | 1+ | 6-10 RBC, 0-1 WBC, rare transitional | n/a | n/a | n/a |
| 16 | Russian Blue | FS | 12.8 | 120.1 | 0.1066 | 256 | 15.8 | 16 | 1.7 | 5.5 | 1.014 | 6 | 0 | 0-2 WBC, 10-15 RBC | n/a | 0.1 | 135 |
| 14 | DSH | MN | 26.4 | 108.4 | 0.2435 | 127 | 6.8 | 18 | 1.07 | 4.2 | 1.02 | 6 | 0 | Inactive | n/a | n/a | 110 |
| 15 | DLH | FS | 12.2 | 114.8 | 0.1063 | 133 | 19.1 | 18 | 1.61 | 3.3 | 1.021 | 7 | 0 | Inactive | n/a | n/a | n/a |
| 17 | DSH | MN | 11.5 | 120 | 0.0958 | 204 | 16.3 | 14 | 1 | 3.8 | 1.015 | 5.5 | 0 | Rare WBCs and RBCs | n/a | n/a | n/a |
| 12 | DSH | FS | 4.6 | 122.7 | 0.0375 | 218 | 14.4 | 24 | 1.5 | 4.9 | 1.015 | 6 | 3+ | Rare RBCs and WBCs | n/a | n/a | n/a |
| 10 | DSH | FS | 16.8 | 125.2 | 0.1342 | 409 | 24.8 | 28 | 2.07 | 3.2 | 1.016 | 6.5 | 0 | Inactive | n/a | n/a | n/a |
| 10 | DSH | FS | 45.6 | 135.7 | 0.3360 | 269 | 17.5 | 23 | 1.3 | 4.4 | 1.016 | 6.5 | Tr | Inactive | NG | n/a | n/a |
| 12 | DSH | MN | 11.1 | 121.2 | 0.0916 | 153 | 15.3 | 13 | 1.6 | 5.9 | 1.014 | 5.5 | 0 | Moderate RBCs, rare WBCs | n/a | 0.2 | n/a |
| 4 | DSH | MN | 31.5 | 115.7 | 0.2723 | 180 | 12.9 | 13 | 1.4 | 4.1 | 1.018 | 6 | 1+ | Rare transitional cells | n/a | 0.3 | 135 |
| 12 | DSH | MN | 19.7 | 128.9 | 0.1528 | 301 | 23 | 19 | 2.3 | 4.3 | 1.016 | 5.5 | Trace | 0-2 WBC | n/a | n/a | 130 |
| 12 | DSH | MN | 9.8 | 116.2 | 0.0843 | 526 | 37 | 24 | 4.1 | 5.3 | 1.015 | 6.5 | 0 | Rare WBCs and Squamous cells | n/a | 0.2 | 160 |
| 16 | DLH | FS | 11.6 | 128.1 | 0.0906 | 406 | 27.4 | 20 | 1.8 | 4.5 | 1.013 | 7 | 2+ | 0-2 RBC, 0-2 WBC | n/a | 0.4 | 120 |
| 13 | DSH | FS | 10.6 | 126 | 0.0841 | 285 | 15.3 | 29 | 1.5 | 5.9 | 1.014 | 5.5 | 0 | Rare transitional cells | NG | 0.1 | 130 |
| 11 | DSH | FS | 59.7 | 131.3 | 0.4547 | 372 | 20.2 | 24 | 1.7 | 3.5 | 1.015 | 6 | 1+ | Inactive | Enterococcus | n/a | 135 |
| 17 | Siamese X | MN | 22.7 | 122.5 | 0.1853 | 217 | 13.5 | 18 | 1.9 | 5.4 | 1.019 | 6.5 | 2+ | Moderate RBCs, rare WBCs and trans | n/a | n/a | 175 |
| 15 | DMH | FS | 35.6 | 119.3 | 0.2984 | 164 | 16.3 | 23 | 2.8 | 5 | 1.013 | 6.5 | 0 | Inactive | NG | n/a | 110 |
| 14 | DMH | FS | 23 | 124.3 | 0.1850 | 248 | 18.1 | 24 | 1.9 | 5 | 1.015 | 6.5 | Trace | Trace RBC/WBC, rare CaOx | n/a | n/a | n/a |
| 11 | DSH | FS | 20.6 | 122.5 | 0.1682 | 150 | 8.9 | 9 | 1.8 | 4.8 | 1.02 | 7.5 | Tr | Moderate RBCs | n/a | 1 | n/a |
| 15 | DSH | MN | 31.8 | 112.9 | 0.2817 | 332 | 16.1 | 23 | 1.7 | 5.2 | 1.015 | 5.5 | Tr | Rare RBCs, transitionals | n/a | n/a | n/a |
| 14 | DSH | FS | 20.5 | 122.4 | 0.1675 | 201 | 11.4 | 15 | 1.1 | 4.4 | 1.013 | 6.5 | 0 | 0-2 WBC, 2-5 RBC | n/a | 0.4 | n/a |
| 16 | Ragdoll | FS | 6.9 | 127 | 0.0543 | 128 | 8.6 | 11 | 1.2 | 4.3 | 1.011 | 6 | 2+ | Rare epithelial cells | n/a | n/a | 140 |
| 15 | Siamese | FS | 31.6 | 125.8 | 0.2512 | 134 | 10.1 | 15 | 1.6 | 5.2 | 1.018 | 5 | 1+ | Inactive | n/a | n/a | n/a |
| 13 | DSH | MN | 5 | 124.2 | 0.0403 | 1115 | 77.6 | 54 | 5.5 | 4.8 | 1.012 | 5 | 0 | Inactive | n/a | n/a | n/a |
| 21 | DSH | MN | 47 | 116.3 | 0.4041 | 146 | 13.5 | 14 | 1.3 | 5.1 | 1.025 | 5.5 | 3+ | Rare WBCs and RBCs | n/a | 0.2 | n/a |
| 8 | DSH | MN | 23.2 | 112.9 | 0.2055 | 188 | 16 | 13 | 1.42 | 5 | 1.026 | 5.5 | 3+ | Rare granular casts | n/a | n/a | n/a |
| 15 | DSH | MN | 13.3 | 126.5 | 0.1051 | 130 | 12.8 | 16 | 1.6 | 5 | 1.013 | 5.5 | 0 | Rare struvite | n/a | n/a | 125 |
| 16 | Persian X | MN | 7.5 | 110.8 | 0.0677 | 320 | 30.8 | 20 | 2.8 | 3 | 1.016 | 6 | 0 | Inactive | NG | n/a | n/a |
| 14 | DSH | FS | 12.6 | 127.2 | 0.0991 | 140 | 11.2 | 12 | 1.3 | 4.3 | 1.019 | 5 | 3+ | Inactive | n/a | 0.7 | 180 |
| 14 | Himalayan | FS | 18.6 | 111 | 0.1676 | 146 | 7.4 | 12 | 1.8 | 4.6 | 1.015 | 5 | 1+ | 0-2 WBC, 1-5 RBC | n/a | n/a | n/a |
| 16 | Snowshoe | FS | 19.7 | 127.5 | 0.1545 | 169 | 11.6 | 11 | 1.6 | 4.9 | 1.025 | 6.5 | Tr | Occasional RBCs and struvite | n/a | n/a | n/a |
| 17 | DSH | MN | 23.6 | 119.6 | 0.1973 | 187 | 18.8 | 17 | 1.7 | 4.7 | 1.025 | 5.5 | Trace | 1-5 RBCs, rare struvite | n/a | n/a | 100 |
| 16 | DSH | FS | 31.4 | 115.9 | 0.2709 | 368 | 30 | 18 | 1.6 | 4.7 | 1.015 | 6 | 3+ | Inactive | n/a | n/a | n/a |
| 15 | DSH | MN | 25.2 | 123.6 | 0.2039 | 199 | 15.7 | 15 | 1.4 | 4.3 | 1.016 | 6 | 0 | Rare RBCs, WBCs, transitionals | n/a | n/a | n/a |
| 13 | DSH | MN | 8.8 | 87.6 | 0.1005 | 148 | 13.3 | 11 | 1.4 | 4.6 | 1.017 | 5.5 | 0 | Inactive | n/a | n/a | 160 |
| 14 | DSH | MN | 36.5 | 114.9 | 0.3177 | 483 | 27.3 | 23 | 1.6 | 4.8 | 1.015 | 5.5 | 1+ | Rare WBCs and RBCs | n/a | n/a | 150 |
| 15 | Siamese X | FS | 17 | 125.6 | 0.1354 | 216 | 18.6 | 17 | 1.3 | 4.3 | 1.015 | 6 | 0 | Inactive | n/a | n/a | 180 |
| 12 | Birman | MN | 25.4 | 110.2 | 0.2305 | 276 | 15 | 20 | 1.4 | 4.7 | 1.021 | 6.5 | 0 | Rare RBCs, WBCs | n/a | n/a | 130 |
| 15 | DSH | FS | 25 | 112.5 | 0.2222 | 192 | 13.3 | 18 | 1.3 | 4.5 | 1.014 | 6.5 | 0 | Inactive | n/a | 0.4 | 200 |
| 16 | DSH | FS | 14.7 | 120.6 | 0.1219 | 184 | 18.2 | 14 | 1.3 | 3.9 | 1.015 | 5 | Tr | 5-10 RBC, 2-5 WBC | NG | 2.2 | 130 |
| 18 | DSH | FS | 29.1 | 119.9 | 0.2427 | 132 | 14.9 | 21 | 1.5 | 4.5 | 1.025 | 5.5 | 2+ | Inactive | NG | n/a | n/a |
| 17 | DSH | MN | 24.9 | 119.4 | 0.2085 | 217 | 13.3 | 12 | 1.6 | 4.3 | 1.014 | 5.5 | 0 | Inactive | n/a | n/a | 75 |
| 15 | DSH | MN | 15.9 | 120.2 | 0.1323 | 296 | 23.1 | 20 | 1.4 | 4.7 | 1.014 | 6.5 | 1+ | Moderate RBCs and rare WBCs | n/a | n/a | n/a |
| 14 | DSH | FS | 12.5 | 125.2 | 0.0998 | 198 | 21.5 | 15 | 1.5 | 5.6 | 1.012 | 8 | Tr | 0-2 WBCs | n/a | 0.2 | n/a |
| 19 | DSH | FS | 16.1 | 118.7 | 0.1356 | 246 | 27.1 | 15 | 1.3 | 5.5 | 1.021 | 7 | Tr | 0-2 WBCs | n/a | n/a | n/a |
| 15 | DSH | FS | 24.7 | 124.2 | 0.1989 | 139 | 17.7 | 16 | 1.7 | 5 | 1.019 | 5.5 | Tr | 0-2 RBC, 0-2 WBC | NG | 0.2 | n/a |
| 16 | DSH | MN | 26.7 | 110.8 | 0.2410 | 223 | 15.1 | 17 | 1.4 | 5.4 | 1.016 | 6 | 0 | Occasional WBCs and RBCS | NG | n/a | 170 |
| 18 | DSH | MN | 19.6 | 108.9 | 0.1800 | 191 | 17.6 | 25 | 1.98 | 4.8 | 1.019 | 5 | Tr | 0-2 WBCs | n/a | 0.1 | 135 |
| 3 | DSH | MN | 26.9 | 116.1 | 0.2317 | 365 | 17.9 |  |  |  | 1.013 |  |  | Lipid droplets | n/a | n/a | n/a |
| 16 | DLH | MN | 9.2 | 117.5 | 0.0783 | 510 | 22.8 | 21 | 2.3 | 4.2 | 1.014 | 6.5 | 0 | 0-2 RBCs and WBCs | n/a | n/a | n/a |
| 5 | Ragdoll | MN | 18.7 | 126.1 | 0.1483 | 830 | 45.1 | 32 | 3.6 | 3.1 | 1.025 | 7 | 3+ | 20-30 RBCs | n/a | 0.1 | 150 |
| 13 | DSH | MN | 54 | 132.4 | 0.4079 | 550 | 46 | 29 | 2.6 | 4.9 | 1.022 | 7 | 3+ | Rare WBCs and RBCs | n/a | 0.1 | 160 |
| 10 | Manx | MN | 17.9 | 124.4 | 0.1439 | 722 | 26.4 | 41 | 2.8 | 3.9 | 1.029 | 6 | 2+ | Rare WBCs and RBCs | n/a | n/a | n/a |

Supplemental Table 1: Biochemical, signalment, and urinalysis parameters for cats enrolled in a study on urinary KIM-1 values in cats with chronic kidney disease. NG: No Growth; n/a: Not applicable
